# The role of all healthcare professionals in cessation

**DOI:** 10.18332/tid/144766

**Published:** 2022-01-17

**Authors:** Jazmin Devonish, Charles Debnam, Elizabeth Furgurson, Hannah Sawa, Carrie Dahlquist, Megan Arendt

**Affiliations:** 1 Tobacco Control Network, District of Columbia Department of Health, Washington, United States; 2 Community Wellness Alliance, Washington, United States; 3 DC Tobacco-Free Coalition, Washington, United States; 4 Action on Smoking and Health, Washington, United States

**Keywords:** tobacco cessation, healthcare, healthcare workers, quitline, healthcare provider

Cigarette smoking rates have been declining across the US, but coordinated efforts across all sectors of public health are still needed to drive down prevalence. To spearhead this message of collaboration, the District of Columbia (D.C.) Department of Health and the DC Tobacco-Free Coalition work with local partners to raise awareness of the harmful effects of tobacco use and to highlight resources available to help smokers quit through a week-long advocacy initiative called DC ‘Call it Quits!’ Week.

As of 2019, the US rate for smoking was 16% compared to the Washington, D.C., rate of 12.7%^1^. Among current smokers in D.C., 62.8% stopped smoking for at least one day, showing that the majority of residents who smoke want to quit^2^. While the overall smoking rate in D.C. is declining, smoking rates are disproportionately high among certain populations, including non-Hispanic African-Americans/Blacks (AA/Blacks), whose prevalence of smoking is three times that of non-Hispanic Whites (Whites). This disparity in use is linked to a person’s environment, which can influence initiation and continuation of smoking. Studies have shown tobacco companies’ marketing tactics target low-income communities in the District. There are ten times as many tobacco advertisements featured in predominantly AA/Black neighborhoods compared to those in predominantly White neighborhoods^3^. This contributes to normalizing smoking and making it more socially acceptable.

Disparities in smoking rates contribute to disparities in health outcomes in the District. Tobacco use, the leading cause of preventable death in the US, increases the risk of chronic diseases including cardiovascular disease, many types of cancer, chronic obstructive pulmonary disease, and diabetes^4^. In D.C., AA/Blacks have higher rates of morbidity and mortality than Whites for cardiovascular disease, lung cancer, colorectal cancer, diabetes, and high blood pressure^2,5^. Additionally, non-smokers are vulnerable to the effects of tobacco through secondhand smoke exposure. Secondhand smoke exposure increases risk for stroke, lung cancer, and coronary heart disease in adults, and for sudden infant death syndrome and acute respiratory infections, among other conditions, in children^6^.

Quitting tobacco can be one of the most important things people can do to improve their health, but it can be challenging because of the addictiveness of nicotine. While almost 70% of smokers in the US want to quit, fewer than a third who try to quit use evidence-based cessation counseling or FDA-approved medications^7^. Cessation has traditionally been shortchanged in tobacco control. In earlier eras, people who smoked were deemed the problem, rather than the victims. Fortunately, this is changing. There are two opportunities for tobacco prevention – stopping youth initiation and adult cessation. Cessation has an immediate, measurable impact. As a trusted source of health information, healthcare providers can play a key role in supporting cessation by not only encouraging cessation but also prescribing drug therapies and referring patients to counseling. The US Preventive Services Task Force recommends clinicians’ assessment for and treatment of tobacco use as a grade A intervention^8^. Unfortunately, fewer than 40% of smokers report being assessed for tobacco use by their healthcare provider^7^. To guide healthcare providers to integrate tobacco conversations and tobacco cessation into their practice, Action on Smoking and Health, the DC Tobacco-Free Coalition, and DC Health collaborated to develop an informational webinar on proven strategies to support cessation, *The Role of All Healthcare Professionals in Cessation*. The webinar provides background on tobacco use in the District, presents an overview of the Ask, Advise and Refer intervention, and DC Quitline services, and highlights successful implementation in a federally qualified health center (FQHC). Cessation is a vital component of ending the tobacco epidemic and DC’s efforts should serve as an example for other jurisdictions.

The initial guidelines, created in 1988 and then updated in 2008, used the 5As (Ask, Advise, Assess, Assist, Arrange). The Ask, Advise, Refer model, based on the 5As, recognizes limited time between a physician and patient to address all health concerns. When possible, referring patients to external sources, such as a state-based quitline service and other community resources, is beneficial.

A state-based quitline coach will assess readiness and available no-cost local resources, assist in determining needs, provide cessation guidance and support, and arrange multiple follow-ups. The DC Quitline, operated by Optum, is provided at no-cost to DC residents by DC Health. While a provider can refer a patient directly to the DC Quitline, residents can also call 1-800-QUIT-NOW for support with their quit attempt. For DC residents, there is also the option to enroll through a web portal, *www.DCQuitNow.com*. The DC Quitline offers three service options tailored to the individual’s needs. The integrated program offers the full menu of cessation services, which includes counseling services with a Quit Coach by phone, texts to help stay on track, FDA-recommended nicotine replacement therapy (NRT), and an interactive web program. The web-only program provides these services minus phone coaching but can be transitioned to the integrated program if desired. The third option is individual services, an *a la carte* menu that offers to individuals options to select which services they want. Among District residents, there is the highest level of participation in the integrated and web-only programs. The DC Quitline is a crucial resource for District residents working toward tobacco cessation.

La Clínica del Pueblo, a federally qualified health center in Washington, DC, and in Maryland, has been working closely with their behavioral health and medical teams to best support their patients who are struggling with tobacco use. The expectations at the health center are to assess smoking status at the first visit and update that status annually; the center stores their documents in *eClinicalWorks*, a centralized electronic health record system. The center uses the Ask, Advise, Refer model, and offers medication, referrals to behavioral health specialists, or referrals to the DC Quitline. The centralized system through the eClinicalWorks portal allows the center to have cohesive documentation of their patients’ tobacco history.

People who smoke are not the perpetrators of the tobacco epidemic. They are the victims. They should be seen as our clients, and everything we do from a policy standpoint should take them into account. With public health organizations, local government, and healthcare providers working together, communities can offer strong support to residents who are struggling with tobacco use. Encouraging cessation initiatives saves lives, strengthens communities, and conserves resources on the individual and societal levels. Cessation is a human rights and health equity issue. As a society, we allowed the tobacco industry to prey on children, particularly among certain populations that already suffer from inequity. Governments have a legal and moral duty to help people quit. Coordination and public awareness of free cessation services are key to reducing tobacco use prevalence and improving long-term health outcomes for all residents.

## Data Availability

Data sharing is not applicable to this article as no new data were created.
